# Supplementary material for: Allosteric regulation alters carrier domain translocation in pyruvate carboxylase
Source: Nat Commun. 2018 Apr 11;9:1384. doi: 10.1038/s41467-018-03814-8 (PMC5895798; doi:10.1038/s41467-018-03814-8)
Supplement: Supplementary file 1 — Supplementary Information [file 41467_2018_3814_MOESM1_ESM.pdf]

Supplementary Information

Allosteric regulation alters carrier domain translocation in pyruvate carboxylase

Liu et al.

Supplementary Table 1. Design of mutations used to generate hybrid tetramers of AnPC and RePC

PC individual domains	Residues		Proposed Function	Mutations	
	RePC	AnPC		RePC	AnPC
Biotin carboxylase domain	E218	E256	MgATP binding and cleavage	E218A	E256A
	E305	E342	General base to deprotonate HCO_3^-	E305Q	E342Q
Carboxyltransferase domain	T882	T923	proton shuttle between carboxybiotin and pyruvate	T882A	T923A
	R621	R662	Pyruvate binding	R621A	R662A
Biotin carboxyl carrier protein domain	K1119	K1171	Biotin attachment site	K1119Q	K1171Q

Supplementary Table 2. Pyruvate carboxylation activity recovered from hybrid tetramers generated by re-mixing and co-expression

**PC homotetramers**	[a] **RePC + 0.25 mM acetyl coenzyme A**			[a] **AnPC + 0.25 mM acetyl coenzyme A**		
	k_{cat} **(min^{-1})**	k_{cat}^{mixing} **(min^{-1})**[b]	k_{cat}^{coexp} **(min^{-1})**[c]	k_{cat} **(min^{-1})**	k_{cat}^{mixing} **(min^{-1})**[b]	k_{cat}^{coexp} **(min^{-1})**[c]
xCT	0.05 ± 0.01	43 ± 2	320 ± 4	0.39 ± 0.07	4.1 ± 0.5	102 ± 6
xBCxBCCP	0.8 ± 0.3			3.1 ± 0.4		
xBC	0.17 ± 0.05	0.8 ± 0.3	12 ± 1	0.16 ± 0.08	0.6 ± 0.1	32 ± 1
xCTxBCCP	1.7 ± 0.4			0.33 ± 0.04		
xBCCP	0.16 ± 0.25		10 ± 1	1.8 ± 0.7	2.6 ± 0.3	9.9 ± 0.4
xBCxCT	0.21 ± 0.12			0.99 ± 0.26		

[a] k_{cat} values were measured in the presence of 25 mM $NaHCO_3$, 2.5 mM MgATP, 12 mM pyruvate and 0.25 mM acetyl CoA. The reported values are the average of 3 independent measurements from one sample. Errors are reported as the standard deviation.
[b] k_{cat} of the PC hybrid tetramers composed of two PC mutants individually purified and mixed together at a 1:1 ratio
[c] k_{cat} of the PC hybrid tetramers purified from the co-expression of two PC mutants in *E. coli*

Supplementary Table 3. Design of the co-expression constructs for generating hybrid tetramers of AnPC and RePC

Co-expression constructs	Domain inactive mutants	Vector	insertion site	N-terminal tag
RePC ×BC/×CT×BCCP	RePC ×BC	pETDuet-1	XbaI/NotI	His_9
	RePC ×CT×BCCP		NdeI/PacI	His_9
RePC ×CT/×BC×BCCP	RePC ×CT	pETDuet-1	XbaI/NotI	His_9
	RePC ×BC×BCCP		NdeI/PacI	His_9
RePC ×BCCP/×BC×CT	RePC ×BCCP	pETDuet-1	XbaI/NotI	His_9
	RePC ×BC×CT		NdeI/PacI	His_9
RePC wild type/×BC×CT×BCCP	RePC wild type	pETDuet-1	XbaI/NotI	-
	RePC ×BC×CT×BCCP		NdeI/PacI	His_9
AnPC ×BC/×CT×BCCP	AnPC ×BC	pRSFDuet-1	NcoI/PacI	-
	AnPC ×CT×BCCP	pET-17b	N/A	His_9
AnPC ×CT/×BC×BCCP	AnPC ×CT	pRSFDuet-1	NcoI/PacI	-
	AnPC ×BC×BCCP	pET-17b	N/A	His_9
AnPC ×BCCP/×BC×CT	AnPC ×BC×CT	pRSFDuet-1	NcoI/PacI	-
	AnPC ×BCCP	pET-17b	N/A	His_9
AnPC wild type/×BC×CT×BCCP	AnPC wild type	pETDuet-1	XbaI/NotI	-
	AnPC ×BCCP		NdeI/PacI	His_9

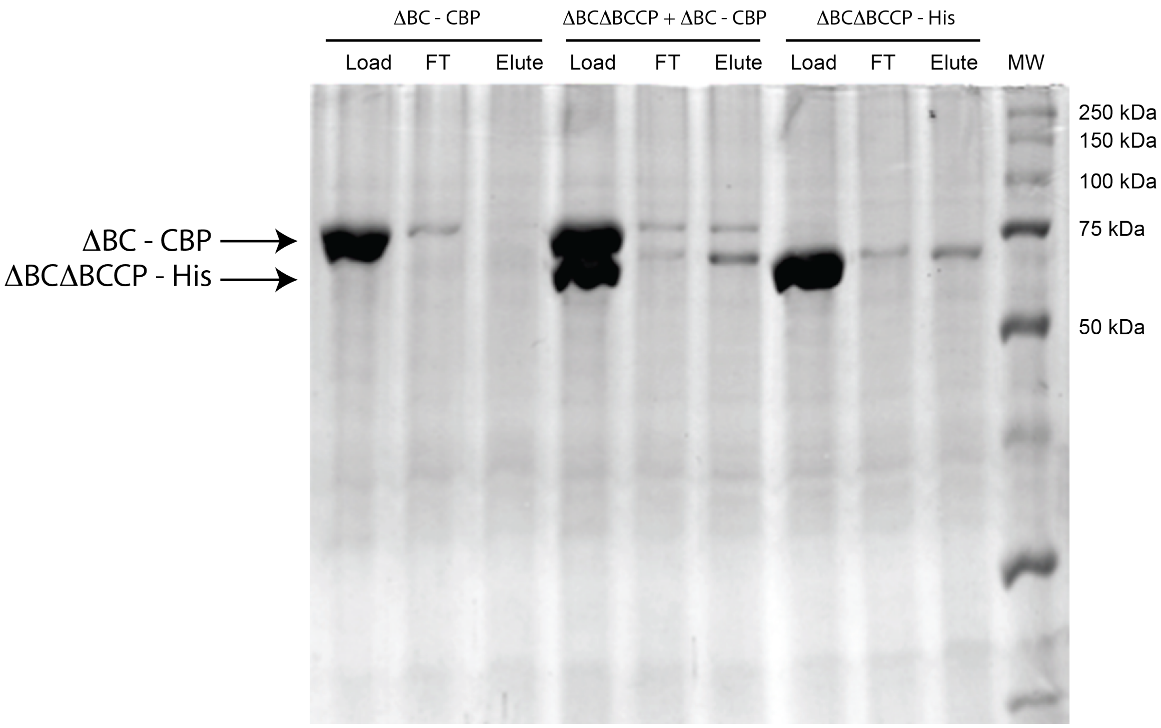

Supplementary Figure 1.
ΔBC RePC dimers are unstable and re-assort into a fully heterogeneous population. ΔBC RePC encoding an N-terminal calmodulin binding peptide affinity tag (ΔBC – CBP) was mixed at a 1:1 molar ratio with ΔBCΔBCCP RePC encoding and N-terminal $(His)_9$ affinity tag (ΔBCΔBCCP - His). The final mixed protein concentration was 1 mg/mL. After incubating at room temperature for 2 hours, the mixture was loaded (Load) onto a gravity-flow Ni^{2+}-affinity column (500 μL resin volume) and the flow through (FT) was collected. The column was washed with 20 column volumes of load buffer before eluting (Elute) with 1 mL elution buffer (see materials and methods for buffer compositions). The SDS-PAGE gel demonstrates that ΔBC – CBP is eluted from the column only when it is mixed with ΔBCΔBCCP-His. Densitometry analysis reveals a 2:1 ratio of ΔBCΔBCCP-His: ΔBC – CBP, which is exactly what is predicted to elute from a fully recombined, heterogeneous population of hybrid dimers.

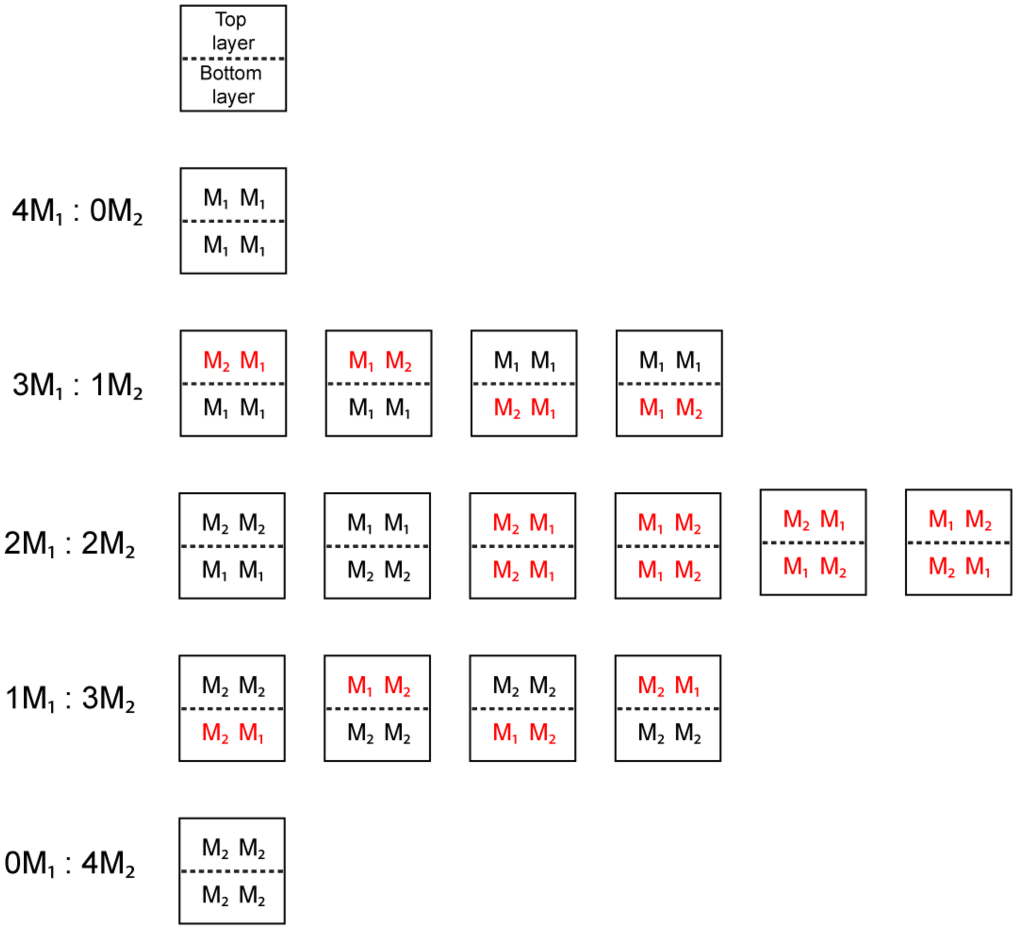

Supplementary Figure 2

The heterogeneous population of tetramers formed through co-expression of two PC full-length genes. M_1, mutant 1; M_2, mutant 2. Assuming the activity is from the catalysis catalyzed by individual domains on the same layer of the tetramer, combination M_1M_1 and M_2M_2 is inactive. M_1M_2 is able to recover activity only if the carrier protein domain is able to adopt certain translocation pathway.

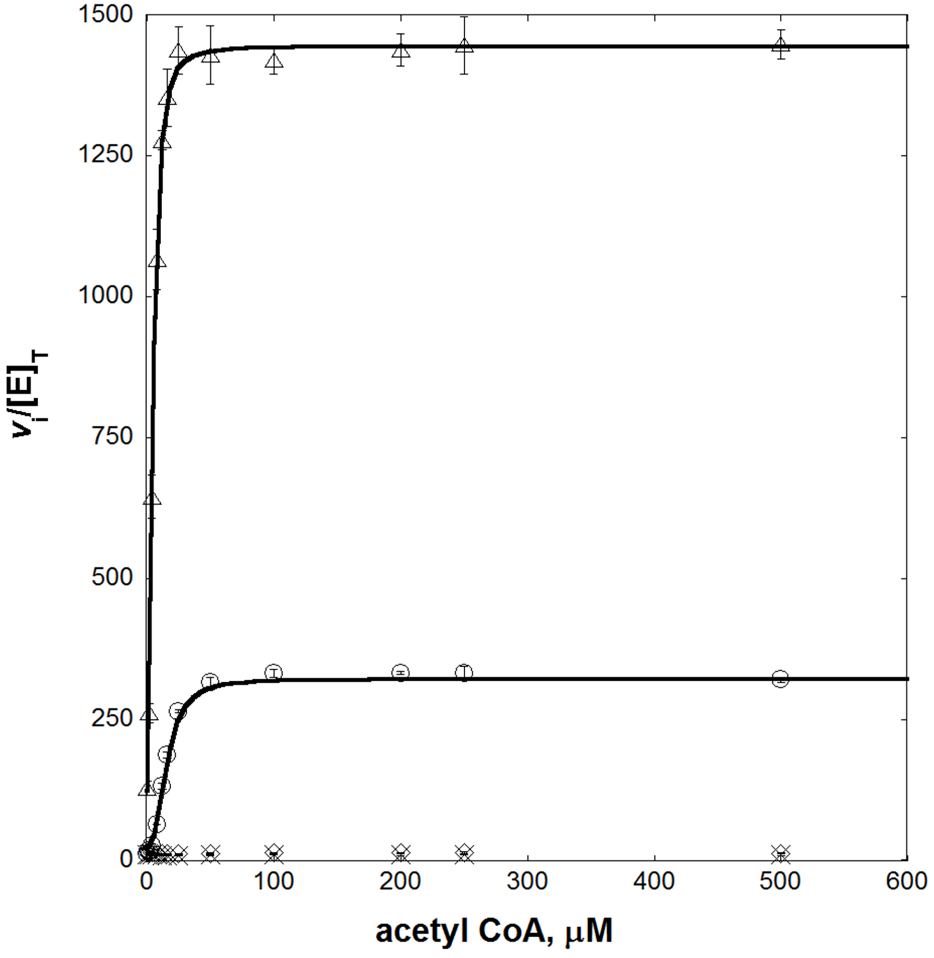

Supplementary Figure 3
Initial velocity as a function of acetyl CoA concentration for wild-type and mixed hybrid tetramers of RePC: wild-type RePC (open triangles), RePC ×CT/×BC×BCCP (open circles), RePC ×BC/×CT×BCCP (open diamonds), RePC ×BCCP/×CT×BC (×). Each independent acetyl CoA concentration was measured 4 independent times. Data points represent average values and error bars represent the standard deviation. These data were used to calculate the values and standard errors for K_a and h reported in Table 2.

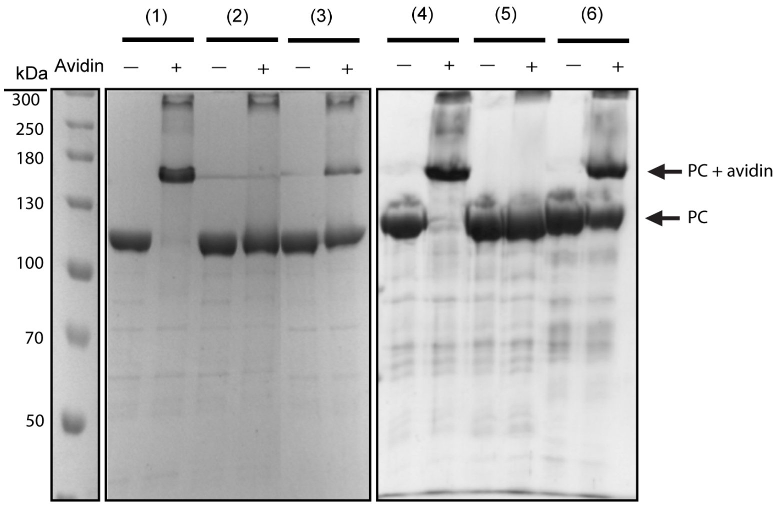

Supplementary Figure 4

Avidin gel shift assay of the control experiment. 1) wild-type RePC; 2) RePC ×BC×CT×BCCP; 3) RePCxBCxCTxBCCP / untagged-RePC wild type; 4) wild-type AnPC ;5) AnPC ×BC×CT×BCCP; 6) AnPCxBCxCTxBCCP / untagged-AnPC wild type

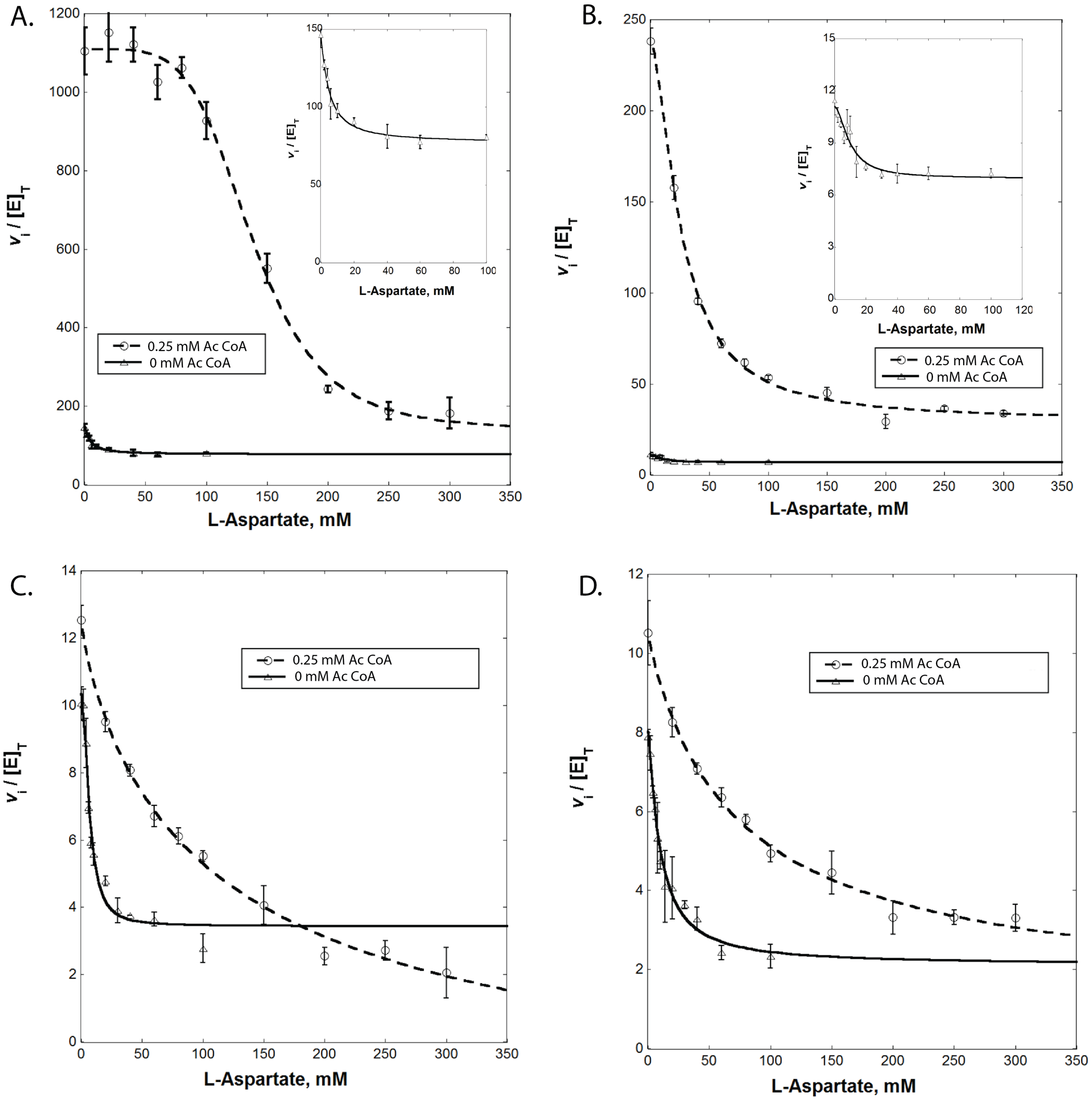

Supplementary Figure 5

Initial velocity as a function of L-aspartate concentration for wild-type and mixed hybrid tetramers of RePC, in the presence and absence of 0.25 mM acetyl CoA. (A) wild-type RePC ± 0.25 mM acetyl CoA (Inset: 0 mM acetyl CoA); (B) RePC ×CT/×BC×BCCP ± 0.25 mM acetyl CoA (Inset: 0 mM acetyl CoA); (C) RePC ×BC/×CT×BCCP ± 0.25 mM acetyl CoA; (D) RePC ×BCCP/×CT×BC ± 0.25 mM acetyl CoA. Each independent acetyl CoA concentration was measured 4 independent times. Data points represent average values and error bars represent the standard deviation. These data were used to calculate the values and standard errors for K_i and h reported in Table 4.

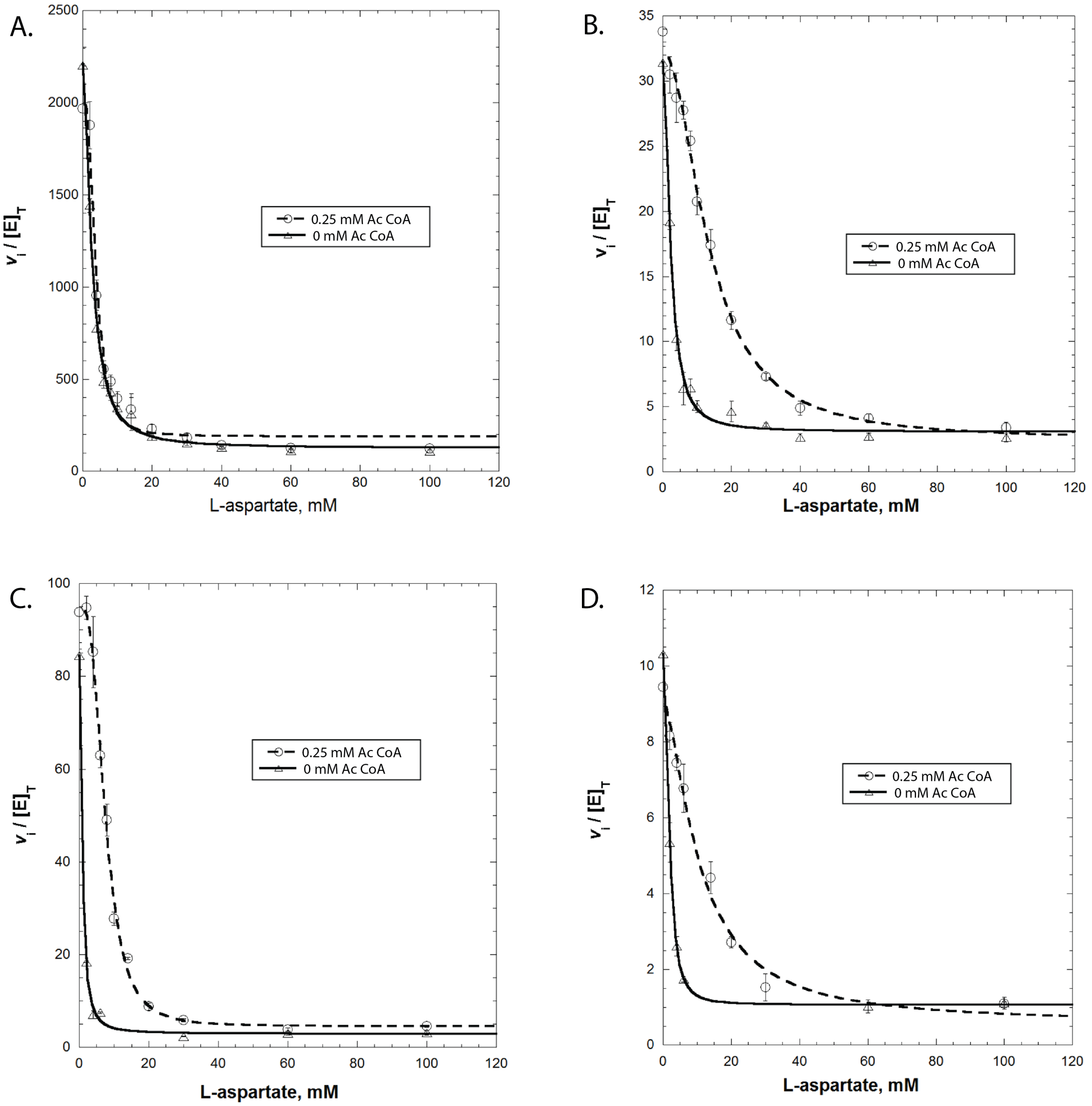

Supplementary Figure 6

Initial velocity as a function of L-aspartate concentration for wild-type and mixed hybrid tetramers of AnPC, in the presence and absence of 0.25 mM acetyl CoA. (A) wild-type AnPC ± 0.25 mM acetyl CoA; (B) RePC ×CT/×BC×BCCP ± 0.25 mM acetyl CoA; (C) RePC ×BC/×CT×BCCP ± 0.25 mM acetyl CoA; (D) RePC ×BCCP/×CT×BC ± 0.25 mM acetyl CoA. Each independent acetyl CoA concentration was measured 4 independent times. Data points represent average values and error bars represent the standard deviation. These data were used to calculate the values and standard errors for K_i and h reported in Table 5.

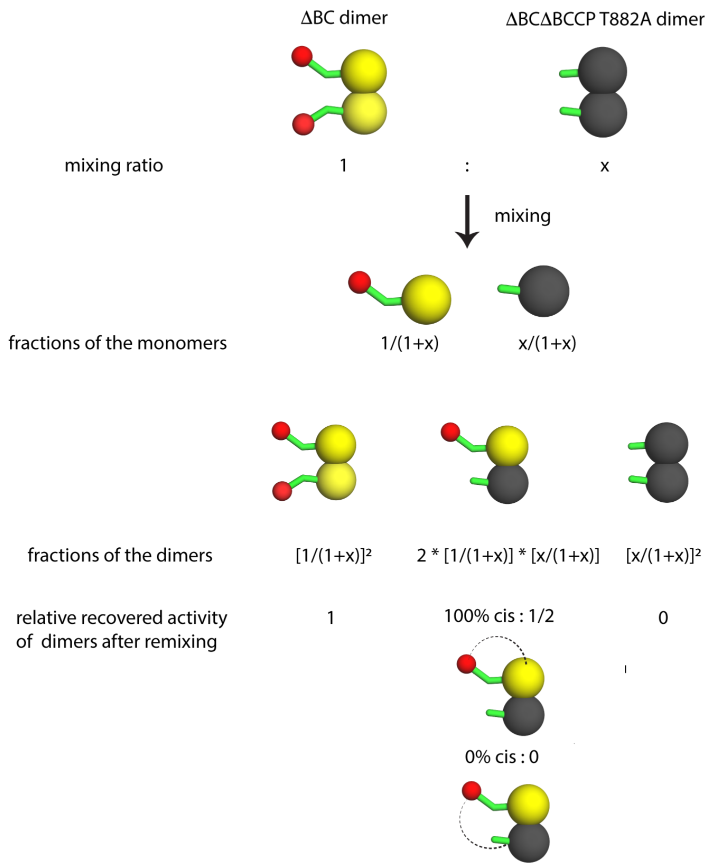

relative recovered activity of the mixture

100% cis :
$$y = 1 * [1/(1+x)]^2 + 2 * [1/(1+x)] * [x/(1+x)] * 1/2 + [x/(1+x)]^2 * 0$$
$$= 1 / (1+x)$$

0% cis :
$$y = 1 * [1/(1+x)]^2 + 0 * [1/(1+x)] * [x/(1+x)] * 1/2 + [x/(1+x)]^2 * 0$$
$$= 1 / (1+x)^2$$

Supplementary Figure 7

Derivation of the equations for the theoretical relative recovered activity of the dimers after remixing.
